# A DFT computational study of the molecular mechanism of [3 + 2] cycloaddition reactions between nitroethene and benzonitrile* N*-oxides

**DOI:** 10.1007/s00894-016-3185-8

**Published:** 2016-12-28

**Authors:** Radomir Jasiński, Ewa Jasińska, Ewa Dresler

**Affiliations:** 1 Institute of Organic Chemistry and Technology, Cracow University of Technology, Warszawska 24, 31-155 Kraków, Poland; 2 Institute of Heavy Organic Synthesis “Blachownia”, Energetyków 9, 47-225 Kędzierzyn-Koźle, Poland

**Keywords:** [3 + 2] cycloaddition, Nitroalkenes, Nitrile* N*-oxides, Mechanism, DFT study

## Abstract

DFT calculations were performed to shed light on the molecular mechanism of [3 + 2] cycloadditions of simple conjugated nitroalkenes to benzonitrile* N*-oxides. In particular, it was found that these processes proceed by a one-step mechanism through asynchronous transition states. According to the latest terminology, they should be considered polar but not stepwise processes.

## Introduction

**Scheme 1:**
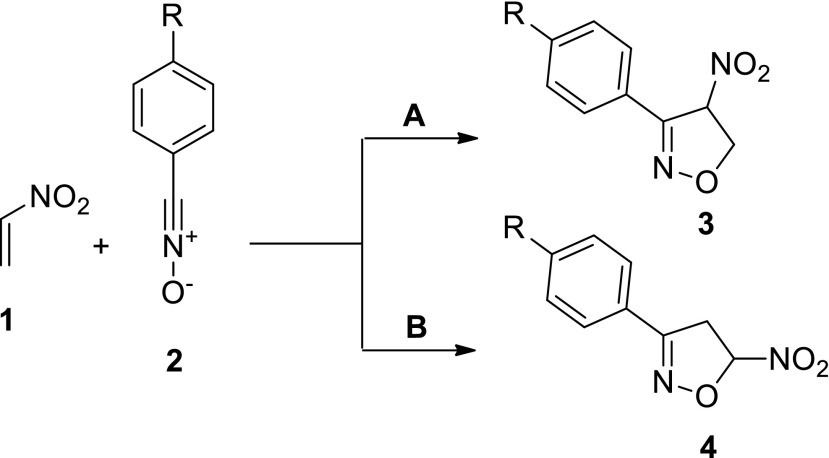
ᅟ

The most versatile method of synthesizing ∆^2^-isoxazolines (3,4-dihydroisoxazoles) is through [3 + 2] cycloaddition reactions between nitrile* N*-oxides (which are allenyl-type three-atom components: TACs [1]) and alkenes [2–6]. The use of nitroalkenes as dipolarophiles in these reactions permits the synthesis of nitro-substituted isoxazolines under mild conditions [5, 7]. These can easily be further functionalized because of their unique tendency to convert the NO_2_ group into other functional groups [7–9]. It is worth mentioning that [3 + 2] cycloadditions of nitrile N-oxides to conjugated nitroalkenes proceed in a highly selective manner. For example, the reaction of benzonitrile* N*-oxide (**1**) (or its aryl-substituted analogs) with nitroethene (**2**) can theoretically proceed along two competing paths, leading to regioisomeric 4- and 5-nitro nitroisoxazolines (**3** and **4**, respectively) (Scheme 1).

In practice, these reactions are realized in a fully regioselective manner, giving high yields of 3-aryl-5-nitroisoxazolines (**4**) [6, 10].

It should be noted at this point that the molecular mechanism of these reactions is not well understood. On the one hand, the literature is often plagued by a belief in a one-step (“pericyclic” [11]) mechanism of [3 + 2] cycloaddition, regardless of the composition of the addent. On the other hand, a number of works challenging this view have been published recently [12–19]. A broad range of mechanisms are now known to occur, proceeding through transition states (TSs) with a range of synchronicities and polarities [1]. Stepwise [3 + 2] cycloaddition is a unique case that can involve zwitterion or diradical formation. Some examples of stepwise [3 + 2] cycloadditions involving conjugated nitroethenes were recently described, including reactions of nitroethene with thiocarbonyl ylides [12] and 1-substituted nitroethenes with diarylnitrones [13, 14]. Additionally, several examples of stepwise cycloadditions involving components other than conjugated nitroalkenes were described; for example, reactions between fluorinated alkenes [15] or phenylisocyanate [16] and* N*-alkylnitrones, dialkyl 2,3-dicyanobut-2-enedioates and azomethine ylides [17], dimethyl 2,3-dicyanofumarate and di(*tert*-butyl)diazomethane [18], as well as methyl nitrile* N*-oxide and tetraaminoethene [19].

**Scheme 2:**
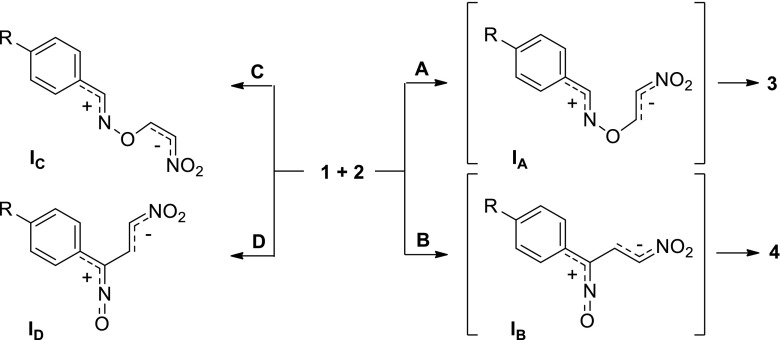
ᅟ

With this in mind, we have found that the molecular mechanism of [3 + 2] cycloadditions of nitrile* N*-oxides to conjugated nitroalkenes requires comprehensive theoretical study using DFT methods. It should be emphasized that the aforementioned reactions have not yet been investigated in this manner, even though such work is important from theoretical and practical points of view. Due to the strong electrophilicity (*ω*) of the conjugated nitroalkenes (*ω* > 1.5 eV [20–22]), it is in fact very likely that these reactions proceed through a zwitterionic intermediate (paths **A** and **B** in Scheme 2). At the same time, it is possible that zwitterionic structures with “extended” conformations are created along the competing paths of the cycloaddition reaction (paths **C** and **D** in Scheme 2). Such a path for addition has recently been analyzed for the reactions of nitroacetylene with a series of allenyl-type TACs [23]. All of the above options deserve detailed consideration.

Recently, we performed theoretical studies of a number of different cycloaddition processes involving conjugated nitroalkenes [24–30], and subsequently carried out experiments examining the reaction selectivity [27, 28, 30–32], activation parameters [33], and kinetic effects of the solvent and substituents [29, 34] for these processes. This comprehensive approach provided good insight into the key properties of the transition states involved. We concluded that DFT calculations should be capable of determining the molecular mechanism of these [3 + 2] cycloadditions. Therefore, in the work reported in the present paper, in order to allow general conclusions to be drawn, benzonitrile* N*-oxide as well as analogs of it containing substituents with different electronic properties were tested as model TACs. In particular, we decided to (a) probe the nature of the interaction between the addents in an elementary cycloaddition step and (b) run simulations of theoretically possible reaction paths of nitroethene with various nitrile* N*-oxides. As an extension of those studies, we also present theoretical studies of similar reactions involving the extremely electrophilic [13] 1,1-dinitroethene molecule here.

## Computational methods

Global reactivity indices (electronic chemical potential* μ*, chemical hardness* η*, global electrophilicity* ω*, global nucleophilicity* N*) were estimated according to the equations recommended by Parr [33] and Domingo [35–37]. In particular, the electronic chemical potentials and chemical hardnesses of the reactants studied here were evaluated in terms of the one-electron energies of the frontier molecular orbitals using the following equations [35, 36]:

$$ \upmu \approx \left({E}_{\mathrm{HOMO}}+{E}_{\mathrm{LUMO}}\right)/2;\kern2.75em \eta \approx {E}_{\mathrm{LUMO}}-{E}_{\mathrm{HOMO}}. $$

The values of* μ* and* η* were then used to calculate* ω* according to the formula

$$ \omega ={\upmu}^2/2\eta . $$

*N* can be expressed [37] as

$$ N={E}_{\mathrm{HOMO}}-{E}_{\mathrm{HOMO}\ \left(\mathrm{tetracyanoethene}\right)}. $$

The local electrophilicity (*ω*_*k*_) [38] concentrated on atom *k* was calculated by projecting the index* ω* onto any reaction center *k* in the molecule using the Parr function P^+^_*k*_:

$$ {\omega}_k={P}_{{}_k}^{+}\cdotp \omega . $$

The local nucleophilicity (*N*_*k*_) [38] concentrated on atom *k* was calculated using the global nucleophilicity* N* and the Parr function P^−^_*k*_ according to the formula

$$ {N}_k={P}_k^{-}\cdotp N. $$

The global and local electronic properties of the reactants considered in this work are collected in Tables 1 and 2.

**Table 1:** Global and local electronic properties of nitroethene (**1**) and its selected 1-substituted derivatives (**5**–**7**)

		R	Global properties				Local properties			
			*μ* (eV)	*η* (eV)	*ω* (eV)	*N* (eV)	P^+^_α_	P^+^_β_	*ω*_α_ (eV)	*ω*_β_ (eV)
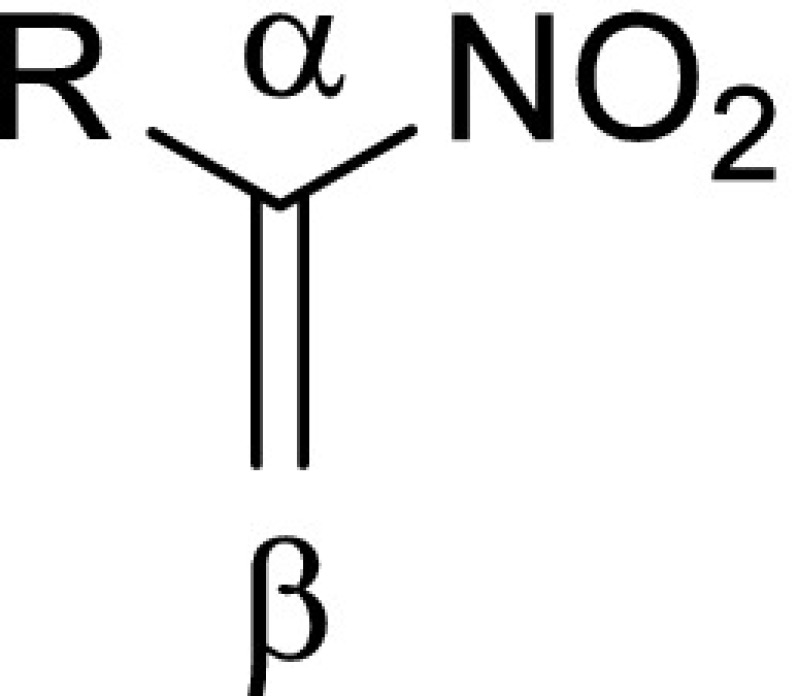	**1**	H	−5.33	5.45	2.61	1.07	0.01	0.44	0.02	1.15
	**5**	Cl	−5.50	5.24	2.88	1.00	0.01	0.45	0.02	1.29
	**6**	COOH	−5.47	5.00	2.99	1.15	0.05	0.64	0.14	1.92
	**7**	NO_2_	−5.98	5.03	3.56	0.62	0.05	0.52	0.18	1.84

**Table 2:** Global and local electronic properties of benzonitrile* N*-oxide and its 4-R-substituted analogs (**2a–h**)

		Substituent		Global properties				Local properties			
		R	*σ*_p_^0^	*μ* (eV)	*η* (eV)	*ω* (eV)	*N* (eV)	P^−^_O_	P^−^_C_	N_O_ (eV)	N_C_ (eV)
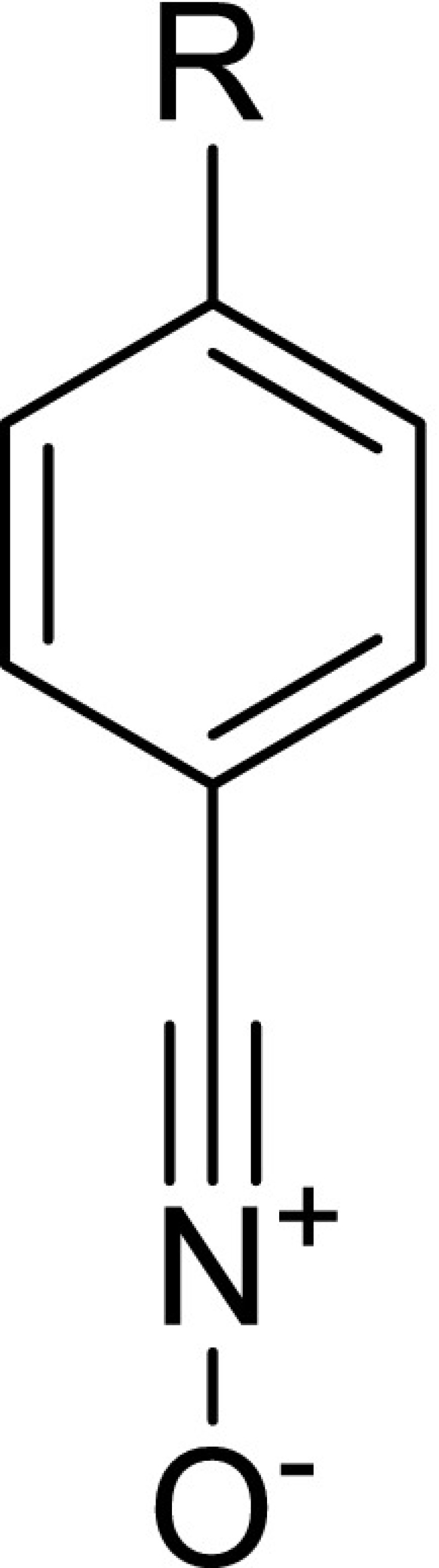	**2a**	NMe_2_	−0.83	−2.95	4.61	0.94	3.87	0.25	0.08	0.98	0.31
	**2b**	OMe	−0.27	−3.42	4.91	1.19	3.24	0.36	0.05	1.16	0.15
	**2c**	Me	−0.17	−3.69	4.97	1.37	2.95	0.42	0.01	1.23	0.04
	**2d**	H	0.00	−3.83	5.02	1.46	2.78	0.54	0.01	1.49	0.03
	**2e**	F	0.06	−3.85	5.02	1.47	2.76	0.43	0.01	1.20	0.03
	**2f**	Ac	0.40	−4.47	4.31	2.32	2.49	0.43	0.01	1.08	0.02
	**2 g**	CN	0.66	−4.63	4.47	2.40	2.25	0.43	0.02	0.96	0.03
	**2 h**	NO_2_	0.78	−5.04	4.03	3.15	2.07	0.48	0.04	0.98	0.09

To localize the transition states (TSs), the hybrid B3LYP functional and the 6-31G(d) basis set included in the GAUSSIAN 09 software package were applied. We subsequently also performed analogous computation at the more advanced B3LYP/6-31 + G(d) and B3LYP/6-311G(d) levels of theory.

The structures corresponding to critical points on the PES along the reaction paths were localized in an analogous manner to that used in a previous analysis of [3 + 2] cycloadditions of (*Z*)-C,N-diphenylnitrone with 1,1-dinitroethene [13]. First-order saddle points were localized using the QST2 and Berny procedures. The transition states were verified by diagonalizing the Hessian matrix and analyzing the intrinsic reaction coordinates (IRC). The polarizable continuum model (PCM) [39], in which the cavity is created as a series of overlapping spheres, was used to calculate solvent effects. Calculations of all critical structures were performed for a temperature* T* = 298 K and pressure* p *= 1 atm. The global electron density transfer (GEDT) [40] was calculated according to the formula

$$ \mathrm{GEDT}=-\varSigma {q}_{\mathrm{A}}, $$

where* q*_A_ is the net Mulliken charge, and the sum is performed over all the atoms of nitroalkene.

Indices of σ-bond development (*l*) were calculated according to the formula [14]

$$ {l}_{\mathrm{A}-\mathrm{B}}=1-\frac{r_{\mathrm{A}-\mathrm{B}}^{\mathrm{TS}}-{\mathrm{r}}_{\mathrm{A}-\mathrm{B}}^{\mathrm{P}}}{r_{\mathrm{A}-\mathrm{B}}^{\mathrm{P}}}, $$

where* r* ^TS^_A–B_ is the distance between the reaction centers A and B at the TS and* r* ^P^_A–B_ is the corresponding distance at the product.

The kinetic parameters as well as important properties of the critical structures are displayed in Tables 3, 4, 5, and 6.

**Table 3:** Eyring parameters for cycloadditions of nitroethene (**1**) to the* N*-oxides **2a**, **2d**, and **2h** according to DFT (PCM) calculations (∆*H*, ∆*G* are in kcal/mol; ∆*S* is in cal/mol K)

Solvent	Level of theory	Reaction	Transition	∆*H*	∆*S*	∆*G*
Toluene	B3LYP/6-31G(d)	**1** + **2a**	**1** + **2a** → **MC**	−3.1	−29.1	5.6
			**1** + **2a** → **TS**_**A**_	10.7	−47.9	25.0
			**1** + **2a** → **3a**	−34.0	−50.5	−19.0
			**1** + **2a** → **TS**_**B**_	11.3	−43.6	24.3
			**1** + **2a** → **4a**	−38.7	−50.3	−23.7
	B3LYP/6-31G(d	**1** + **2d**	**1** + **2d** → **MC**	−3.0	−23.6	4.0
			**1** + **2d** → **TS**_**A**_	12.9	−43.3	25.8
			**1** + **2d** → **3d**	−34.1	−48.7	−19.6
			**1** + **2d** → **TS**_**B**_	12.1	−41.9	24.6
			**1** + **2d** → **4d**	−38.5	−47.8	−24.3
	B3LYP/6-31 + G(d)	**1** + **2d**	**1** + **2d** → **MC**	−1.4	−25.4	6.1
			**1** + **2d** → **TS**_**A**_	14.8	−42.9	27.6
			**1** + **2d** → **3d**	−30.4	−47.5	−16.3
			**1** + **2d** → **TS**_**B**_	14.2	−42.7	26.9
			**1** + **2d** → **4d**	−35.2	−46.1	−21.4
	B3LYP/6-311G(d)	**1** + **2d**	**1** + **2d** → **MC**	−3.0	−28.6	5.5
			**1** + **2d** → **TS**_**A**_	14.6	−44.3	27.8
			**1** + **2d** → **3d**	−29.2	−48.3	−14.8
			**1** + **2d** → **TS**_**B**_	14.0	−41.7	26.5
			**1** + **2d** → **4d**	−33.8	−47.4	−19.7
	B3LYP/6-31G(d)	**1** + **2h**	**1** + **2h** → **MC**	−3.4	−29.3	5.4
			**1** + **2h** → **TS**_**A**_	13.1	−45.1	26.6
			**1** + **2h** → **3 h**	−34.8	−48.5	−20.3
			**1** + **2h** → **TS**_**B**_	12.4	−43.9	25.5
			**1** + **2h** → **4 h**	−38.8	−48.3	−24.3
Water	B3LYP/6-31G(d	**1** + **2d**	**1** + **2d** → **MC**	−1.5	−26.6	6.4
			**1** + **2d** → **TS**_**A**_	13.6	−43.4	26.5
			**1** + **2d** → **3d**	−33.5	−48.2	−19.1
			**1** + **2d** → **TS**_**B**_	12.3	−41.8	24.8
			**1** + **2d** → **4d**	−38.5	−47.3	−24.5

**Table 4:** Eyring parameters for cycloadditions of 1,1-dinitroethene (**7**) to benzonitrile* N*-oxide (**2d**) according to B3LYP/6-31G(d) (PCM) calculations (∆*H*, ∆*G* are in kcal/mol; ∆*S* is in cal/mol K)

Transition	Toluene			Water		
	∆*H*	∆*G*	∆*S*	∆*H*	∆*G*	∆*S*
**7** + **2d** → **MC**_**A**_	−4.2	1.8	−20.1	−2.4	3.3	−18.9
**7** + **2d** → **TS**_**A**_	6.9	17.2	−34.6	7.4	17.5	−34.1
**7** + **2d** → **8**	−35.0	−22.6	−41.5	−32.5	−20.3	−40.8
**7** + **2d** → **MC**_**B**_	−3.6	2.6	−21.0	−2.2	3.4	−19.0
**7** + **2d** → **TS**_**B**_	8.2	18.8	−35.7	8.5	18.4	−33.4
**7** + **2d** → **9**	−42.9	−31.2	−39.1	−41.5	−30.4	−37.5

**Table 5:** Key parameters of critical structures in cycloadditions of nitroethene (**1**) to the* N*-oxides **2a**, **2d**, and **2h** according to B3LYP/6-31G(d) (PCM) calculations

Solvent	Reaction	Structure	C3–C4		C5–O1		∆*l*	*μ*_D_ (D)	GEDT (*e*)
			*r* (Å)	*l*	*r* (Å)	*l*			
Toluene	**1** + **2a**	**MC**	4.896		3.371			4.96	0.00
		**TS**_**A**_	2.356	0.451	2.009	0.615	0.16	8.50	0.12
		**3a**	1.521		1.450			6.01	0.13
		**TS**_**B**_	2.140	0.589	2.316	0.331	0.26	12.34	0.10
		**4a**	1.516		1.387			8.86	0.15
	**1** + **2d**	**MC**	4.459		4.374			2.04	0.00
		**TS**_**A**_	2.317	0.477	2.073	0.571	0.09	5.03	0.05
		**3d**	1.521		1.450			3.54	0.15
		**TS**_**B**_	2.159	0.575	2.362	0.304	0.27	8.25	0.08
		**4d**	1.515		1.393			5.68	0.17
	**1** + **2h**	**MC**	3.830		3.089			4.97	0.00
		**TS**_**A**_	2.306	0.483	2.116	0.546	0.06	5.10	0.02
		**3h**	1.520		1.455			5.98	0.18
		**TS**_**B**_	2.174	0.564	2.370	0.304	0.26	3.86	0.05
		**4h**	1.514		1.398			4.61	0.20
Water	**1** + **2d**	**MC**	4.330		4.420			3.24	0.00
		**TS**_**A**_	2.324	0.472	2.047	0.591	0.12	5.58	0.09
		**3d**	1.522		1.452			4.28	0.18
		**TS**_**B**_	2.140	0.586	2.378	0.293	0.29	9.44	0.09
		**4d**	1.514		1.393			6.52	0.19

**Table 6:** Key parameters of critical structures in the cycloaddition of 1,1-dinitroethene (**7**) to benzonitrile* N*-oxide (**2d**) according to B3LYP/6-31G(d) (PCM) calculations

Solvent	Structure	C3–C4		C5–O1		∆*l*	*μ*_D_ (D)	GEDT (*e*)
		*r* (Å)	*l*	*r* (Å)	*l*			
Toluene	**MC**_**A**_	4.149		2.814			4.14	0.00
	**TS**_**A**_	2.517	0.727	1.835	0.344	0.38	5.48	0.24
	**8**	1.520		1.441			1.74	0.06
	**MC**_**B**_	5.207		3.179			3.96	0.00
	**TS**_**B**_	2.091	0.616	2.387	0.264	0.35	10.38	0.19
	**9**	1.510		1.375			7.60	0.13
Water	**MC**_**A**_	4.119		2.826			4.41	0.00
	**TS**_**A**_	2.634	0.267	1.755	0.784	0.52	7.18	0.30
	**8**	1.520		1.443			1.77	0.07
	**MC**_**B**_	5.288		3.278			4.57	0.00
	**TS**_**B**_	2.065	0.632	2.420	0.238	0.39	11.84	0.21
	**9**	1.510		1.373			8.57	0.14

## Results and discussion

### Analysis of interactions between addents

First, we decided to shed some light on the nature of the interactions between the addents. To do this, we used the electronic properties of the reactants, which were estimated using equations defined based on conceptual density functional theory [41]. A similar approach was successfully used recently to explain the paths followed by a number of different biomolecular processes (see for example [42–45]). In this theory, nitroethene is classified as a strong electrophile (*ω* > 1.5 eV) [36]. Its electrophilicity can be enhanced by introducing a second electron-withdrawing group (EWG) at position 1 of the nitrovinyl fragment. Accordingly, the presence of a Cl substituent increases the value of* ω* for nitroalkene to 2.88 eV, and that of the nitro group to 3.56 eV.

The electronic properties of* N*-oxides of aromatic nitriles vary widely. The global electrophilicity of benzonitrile* N*-oxide **2d** is 1.46 eV, which makes it a moderate electrophile. However, gradually increasing the electron-donating power of a substituent at the 4-position on the* N*-oxide phenyl ring causes a gradual change in its electronic properties. In particular, for the 4-dimethylamino-substituted* N*-oxide **2a**,* ω* is below 1 eV. This means that **2a** will exhibit only marginally electrophilic properties, but will show strong nucleophilic power, as indicated by the value of* N* (>3.8 eV). Replacing the dimethylamino group with a strongly electron-withdrawing nitro group results in a dramatic change in the properties of the* N*-oxide. In particular, the 4-nitro-substituted* N*-oxide **2h** is characterized by strong electrophilic properties (*ω* > 3 eV)—stronger than nitroethene (!).

We then analyzed the local reactivity for different pairs of reactants. We found that the oxygen atom on the CNO fragment is a strongly nucleophilic reaction center in all of the* N*-oxides. In turn, the strongest electrophilic reaction center is always the β atom of the nitrovinyl fragment in the nitroalkenes. If we assume that these centers govern the reaction path, then the products of the cycloadditions should be 4-nitroisoxazolines. However, this conclusion conflicts with the experimental data, because only the corresponding 5-nitroisoxazolines are formed in this process.

It should be noted at this point that only extremely electrophilic addents were considered for further detailed study of the mechanistic aspects of the corresponding cycloadditions. This allowed us to get a good idea of the mechanism associated with the [3 + 2] cycloaddition processes without having to calculate the full reaction paths for all such processes.

### Kinetic aspects

**Fig. 1:**
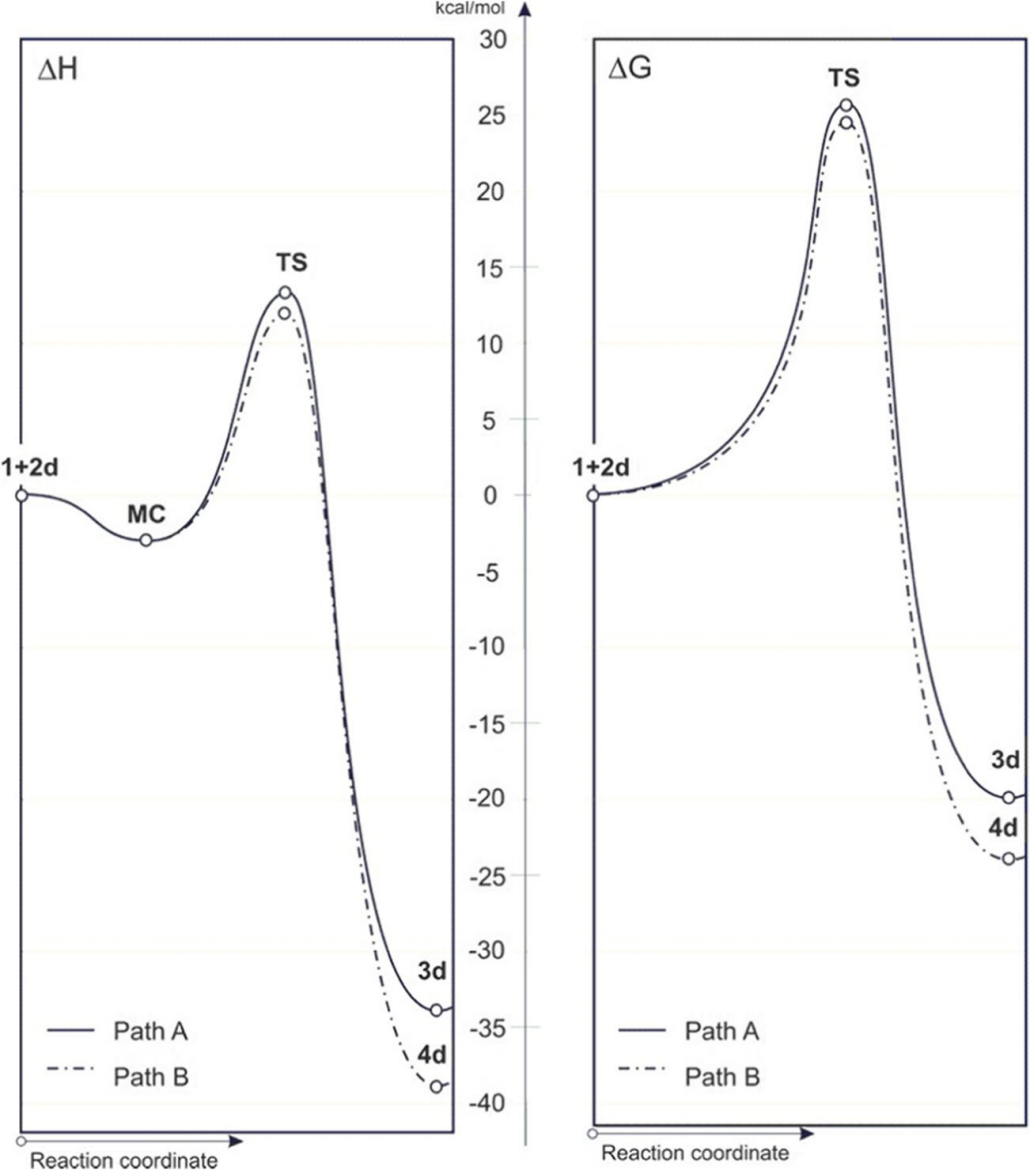
Energetic profiles for cycloaddition between nitroethene (**1**) and benzonitrile* N*-oxide (**2d**) in toluene according to B3LYP/6-31G(d) (PCM) calculations

DFT calculations show that the [3 + 2] cycloaddition of nitroethene (**1**) to* N*-oxide **2d** in weakly polar toluene proceeds by a one-step mechanism (Fig. 1, Table 3). In the first step of the process, the enthalpy of the reacting system decreases due to the formation of a pre-reaction complex (**MC**). Depending on the reaction path, this complex can be converted to regioisomeric isoxazoline **3d** or **4d**. Each of these conversions entails overcoming an activation barrier. The kinetically favored conversion is that associated with path **B**, leading to the formation of a 5-nitro-substituted adduct, which—as shown by experimental studies—does indeed form during this reaction [6, 10]. So, contrary to expectations arising from regioselectivity, based on the analysis of the stationary states of the addends (see the preceding paragraph), a DFT-based exploration of the reaction pathways can accurately determine the regiochemistry of cycloaddition. However, all attempts to find a reaction path that leads to the adduct through an acyclic intermediate were unsuccessful. We also failed to obtain any stable structures of hypothetical zwitterions in an “extended” conformation.

Similar studies were also performed for reactions involving representative* N*-substituted analogs of benzonitrile* N*-oxide. In the calculations of the reaction paths for the parent reaction system involving nitroethene and unsubstituted benzonitrile* N*-oxide, several basis sets were applied (Table 3). It was found that calculations using the simple 6-31G(d) basis set gave practically identical (from a mechanistic point of view) results to calculations using higher levels of theory. In particular, in each instance, DFT calculations indicated a one-step reaction mechanism in which the favored path leads to a product with the nitro group at the 5-position on the heterocyclic ring. Thus, only the B3LYP/6-31G(d) level of theory was applied in subsequent investigations.

We found that, in quantitative terms, the energy profiles for the cycloadditions **1** + **2a** and **1** + **2h** are the same as that for **2d + 1** cycloaddition, although they would be somewhat different quantitatively. In particular, introducing the (electron-donating) dimethylamino substituent at the 4-position of the phenyl ring of the* N*-oxide reduces the activation barrier, whereas the introduction of the (electron-withdrawing) nitro group increases the aforementioned barrier. However, reaction path **B** is always kinetically favored.

We then decided to investigate the effect of solvent polarity on the course of the reaction. We found that replacing toluene with a more polar medium (water) prompts an increase in the activation barrier along both regioisomeric paths.

**Scheme 3:**
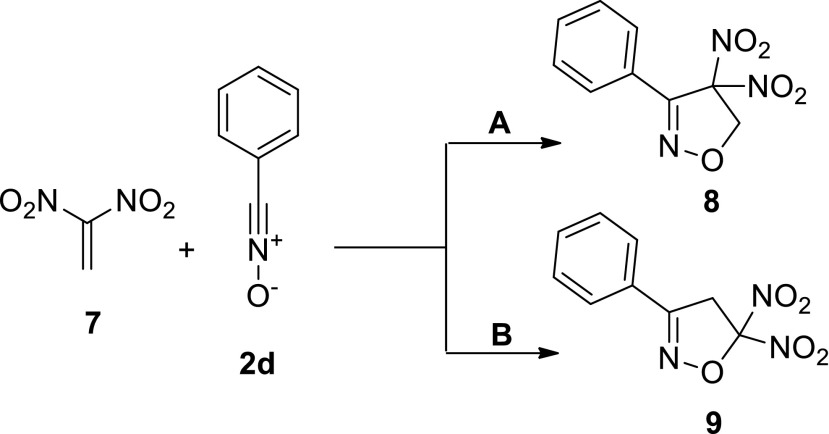
ᅟ

As an extension of our studies, we performed a similar analysis of the energy profile of a similar cycloaddition involving 1,1-dinitroethene **7** and* N*-oxide **2d** (Scheme 3), which has not yet been studied experimentally. Our previous work [13] indicated that nitroalkene **7** will react with* C*,*N*-diphenylnitrone via a stepwise zwitterionic mechanism. As in the case of **1 + 2** cycloaddition, we considered both of the theoretically possible regioisomeric reaction routes (**A** and **B**; see Scheme 3).

The results indicated that, in toluene, the energy profile of **7 + 2d** cycloaddition is qualitatively the same as that of **2d + 1** cycloaddition. However, due to the strongly electrophilic character of nitroalkene **7**, these processes take place much more quickly than in the reaction involving nitroethene **1** (Table 4). The regioselectivity of this cycloaddition is different from that of **1 + 2d** cycloaddition. In particular, from a kinetic point of view, the path leading to the 4-nitro-substituted adduct is favored. All attempts to find paths leading to cycloadducts through an acyclic intermediate step were unsuccesful, as were efforts to find paths leading to hypothetical zwitterions with an “extended” conformation. Very similar results were seen for calculations of **7 + 2d** cycloaddition in the presence of a more polar medium (water).

The studies described above indicate that there is an important difference between the paths of the reactions of model nitriles and imine* N*-oxides with nitroethene **1** and dinitroethene **7**. The former TAC reacts with both of those nitroalkenes (despite their rather different global electrophilicities) by a one-step mechanism, while the latter reacts with nitroethene **1** according to a single-step mechanism but with 1,1-dinitroethene **7** by a two-step zwitterionic mechanism [13].

### Key structures

**Fig. 2:**
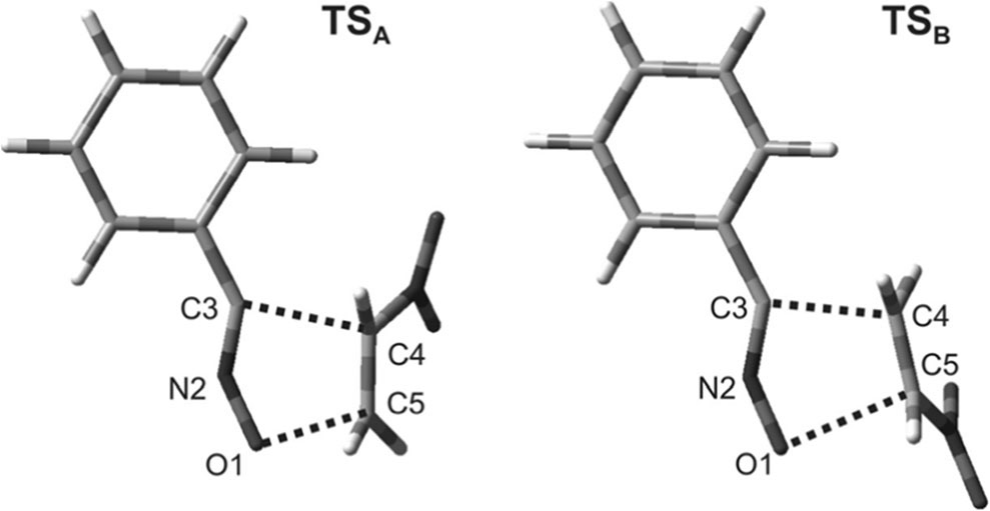
Views of the TSs that occur during the cycloaddition of nitroethene (**1**) to benzonitrile* N*-oxide (**2d**) in toluene, as derived via B3LYP/6-31G(d) (PCM) calculations

B3LYP/6-31G (d) calculations showed that two new σ-bonds always form in both TSs of the **1 + 2d** reaction (Fig. 2). These bonds are formed between the atoms O1 and C5 and between C3 and C4, although the bond with the β carbon of the nitroalkene substructure forms faster. Thus, the structures of the TSs correlate well with the data obtained upon analyzing the local electrophilicity in nitroalkenes. The energetically favored TS along path **B** is even more asynchronous (see the ∆*l* values in Table 5). Both of the TSs considered are polar, as demonstrated by their dipole moments and GEDT values (Table 5).

The synchronicity of **TS**_**A**_ can be controlled to some extent by altering the substituent on the phenyl ring of TAC. In particular, introducing an electron-donating group increases the asynchronicity of the TS, while adding an electron-withdrawing group reduces its asynchronicity. The type of substituent present does not appear to influence the asynchronicity of **TS**_**B**_.

Next, we analyzed the effect of the polarity of the reaction medium on the characteristics of the TSs. We found that replacing the weakly polar solvent toluene with a strongly polar medium (nitromethane or water) increases the asynchronicity of the TSs. At the same time, they increase in polarity. However, these changes are not significant enough to induce a stepwise zwitterionic mechanism.

**Fig. 3:**
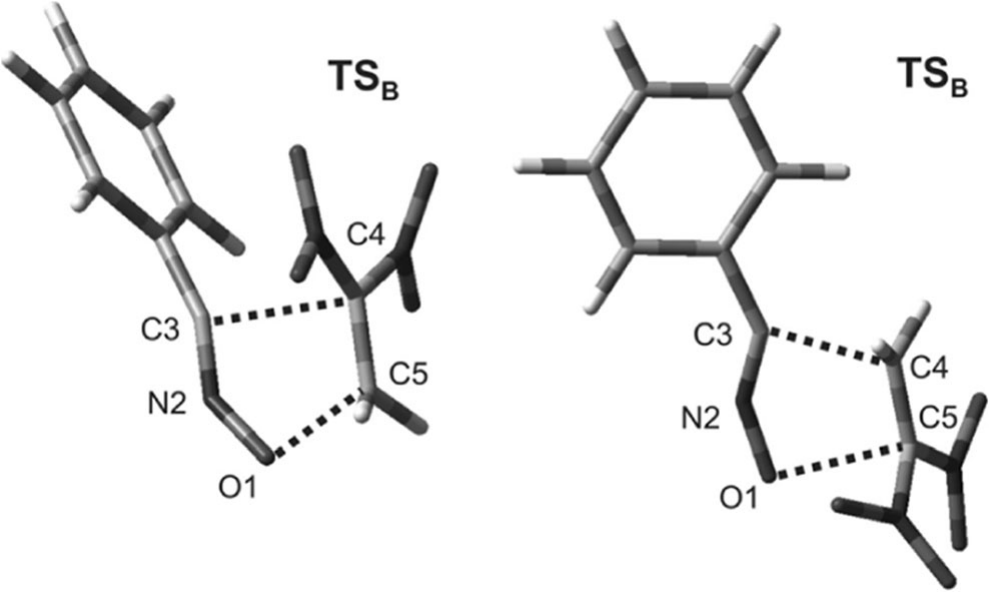
Views of the TSs that occur during the cycloaddition between 1,1-dinitroethene (**7**) and benzonitrile* N*-oxide (**2d**) in toluene, as derived via B3LYP/6-31G(d) (PCM) calculations

A similar analysis was performed for the TSs in the reaction between dinitroethene **7** and TAC **2d**. In accordance with our expectations, these structures were found to be far more asynchronous and strongly polar (Fig. 3, Table 6) than the corresponding TSs in the **1 + 2d** reaction. Our attempts to optimize stable zwitterionic structures that could hypothetically form in the **7 + 2d** reaction failed.

## Conclusions

Regardless of the basis set applied, our B3LYP results clearly indicate that [3 + 2] cycloadditions of simple nitroalkenes to arylonitrile* N*-oxides proceed via a one-step mechanism. According to Domingo’s terminology [43], this mechanism should be interpreted as polar. DFT calculations also showed that the favored reaction path leads to an adduct with a nitro group on C5, which agrees well with experimental observations. The transition-state synchronicity can be controlled to a certain degree by changing the polarity of the reaction medium and the nature of the substituent on the* N*-oxide phenyl ring. However, this is not enough to induce a switch to a two-step reaction mechanism with a zwitterionic intermediate. Nor was such a mechanism found to be possible in an analogous cycloaddition with a much stronger electrophilic component (1,1-dinitroethene).
